# Supplementary material for: Are carbon emissions trading and green financial instruments synergistic? -Comprehensive quantitative research based on content analysis
Source: PLoS One. 2024 Mar 7;19(3):e0298601. doi: 10.1371/journal.pone.0298601 (PMC10919601; doi:10.1371/journal.pone.0298601)
Supplement: S1 Data — (ZIP) [file pone.0298601.s001.zip › Data/地级市Z_F工作报告.zip!/╔╜╢1⁄2/┴─│╟╩╨/└┤╫╘╣1⁄2╓┌║┼í╛┬φ┐╦╩2╛▌═°í┐_2003.docx]

各位代表：

现在，我代表市人民政府，向大会作政府工作报告，请予审议，并请市政协委员和其他列席的同志提出意见。

一、 过去五年工作的回顾

市十三届人大一次会议以来，本届政府在省委、省政府和中共聊城市委的正确领导下，坚持以邓小平理论和“三个代表”重要思想为指导，深入贯彻党的十五大和省七次党代会精神，认真落实市九次党代会和市人大及其常委会的各项决议，解放思想，与时俱进，抢抓机遇，加快发展，全市改革开放和现代化建设迈出了新的步伐，取得了巨大成就。

（一）综合经济实力迈上新台阶。五年来，我们胜利完成了“九五”计划和“十五”计划前两年的任务，国民经济保持持续快速发展，主要经济指标增幅高于全省平均水平，许多方面实现了历史性突破。全市国内生产总值由1997年的217.7亿元增加到2002年的375.5亿元，按可比口径年均增长12.2%，其中一、二、三次产业年均分别增长5％、15.3％和15.4％；人均国内生产总值达到6712元。地方财政收入由8.5亿元增加到16.7亿元，年均增长20. 1%；财政支出由14.5亿元增加到29.4亿元，年均增长15.2%。各类存款、贷款余额由177.4亿元和166. 8亿元增加到338亿元和272.6亿元，分别净增160.5亿元和105.7亿元。五年累计完成固定资产投资438.7亿元，年均增长20.8%。

（二）经济结构调整取得重大进展。五年来，我们坚持把结构调整作为主线，不断调整优化第一产业，加快发展二、三产业，全市三次产业的比例由１９９７年的３7.9∶４１.2∶２0.9调整为２００２年的２５.6∶４6.1∶２８.3。农村产业结构调整迈出重大步伐，粮经作物种植比例由1997年的6.2∶3.8调整为５.２∶４.８，粮食总产量稳定在３０亿公斤以上，优质高效经济作物已形成主导产业，林牧渔业比重提高，农业产业化、市场化进程加快。工业经济保持高速增长，主要指标增幅位居全省前列，一批优势骨干企业已经形成。2002年，全市规模以上工业实现增加值１０６.2亿元、销售收入３５1.9亿元、利税３５.2亿元、利润２1.1亿元，分别比1997年增长2.1倍、1.4倍、1.8倍和2.2倍；全市有５3户企业实现利税超千万元，其中9户企业过亿元。商贸流通业发展步伐加快，旅游业、房地产业已成为新的增长点，全市社会消费品零售总额达到６４.１亿元，比1997年增长63.3%。民营经济发展实现重大突破，提供税收占地方财政收入的比重由1997年的10.4%提高到29.6%，在增加财力、扩大城乡就业方面发挥了重要作用。

（三）各项改革不断深化。五年来，我们积极推进各项改革，社会主义市场经济体制基本框架初步建立。国有经济布局调整有新的进展，国企改革与脱困“三年两个目标”如期实现，多数国有大中型企业初步建立现代企业制度。企业重组和资本运营成效显著，全市有3户企业成为上市公司，直接融资12.6亿元。国有资产管理体制改革快速推进，新型国有资产管理、运营和监督体制框架初步形成。城市资产经营迈出新步伐，公益事业投资多元化实现重大突破。农村产权制度改革走在全省前列，农村税费改革取得重要阶段性成果，农村新型合作经济组织发展加快。财税、金融、外贸、价格、流通、住房等项改革稳步推进。整顿和规范市场经济秩序取得显著成效。政府机构改革顺利完成，政府职能进一步转变，行政效率明显提高。

（四）对外开放实现重大突破。五年来，我们坚定不移地实施外向带动战略，对外开放的新格局基本形成。２００２年，全市合同利用外资首次突破１亿美元，比１９９７年增长68%，实际利用外资6004万美元，比1997年增长67.7%，五年累计利用外资1.55亿美元；进出口总值达到１.９5亿美元，比１９９７年增长40.3%，其中出口创汇１.４亿美元，比１９９７年增长2.5倍。聊城经济开发区建设步伐加快，进区项目达到262个，累计引进国内外资金41.4亿元。县（市、区）相继建立了工业园区，初步构筑起招商引资的平台。对外经济技术交流与合作不断扩大，工程承包、技术合作等都有新的发展。２００１年以来，我们积极实施“大开放、大招商，促进大发展”战略，仅一年多的时间，全市引进国内外投资项目1480个，实际到位资金７５.3亿元，培植了一批新的经济增长点。

（五）城乡面貌发生巨大变化。五年来，我们抓住国家实施积极财政政策和稳健货币政策的有利时机，抓住省委、省政府向西部倾斜的大好机遇，加大了投资力度，重点加强了交通、水利、通信、电力、城市基础设施和工业项目建设。先后完成了邯济铁路、济聊馆高速公路、农村电网改造、彭楼引黄入鲁、中华电力聊城发电厂一号机组等一批基础设施项目和重点工业项目。五年间，全市新建、改建公路719.4公里，新增程控电话交换机71.1万门，新增城镇日供水能力18.2万吨，新增农业有效灌溉面积23万亩，基础设施得到了明显改善。五年来，我们大力实施城市化战略，确立了建设卓越秀美江北水城的战略定位，城市建设步伐明显加快，城市化水平由1997年的16.5%提高到28.1％。五年间，全市城市开发建设累计投资57.2亿元，其中聊城城区31.4亿元，开发建设了东昌湖、古运河、徒骇河等一批靓点工程，新增城市绿地面积372公顷，新增城市道路366.7万平方米，聊城城区实现了集中供热、供天然气，建成了污水处理厂和东阿引供水工程，完成旧城改造拆迁面积334.5万平方米，新增住宅建筑面积276万平方米，城市面貌发生了重大变化。县市城区和小城镇建设步伐加快，农村基本实现了通路、通电和通电话，部分农村居民饮水困难问题得到解决，乡村面貌发生了很大变化。

（六）各项社会事业全面发展。五年来，我们认真实施科教兴聊战略，突出抓了科技、教育的发展，与高等院校和科研院所合作迈出实质性步伐。全市五年累计取得重大科技成果248项，建成高科技农业园（区）120个，培植高新技术企业13家，新增国家和省级技术开发中心7个，有500多家企业与高等院校和科研院所建立了稳固的合作关系。各级各类教育加快发展，提前完成“普九”任务，顺利实现“两基”目标，全市累计有17.5万名初高中毕业生升入大中专院校；农村义务教育管理体制改革成效明显，中小学布局调整、危房改造全面完成；职业教育、成人教育和民办教育进一步发展，建成了聊城职业技术学院，聊城师范学院发展成为综合性的聊城大学。狠抓三项基本国策的落实，计划生育实现了低生育水平，滥占耕地的现象得到有效扼制，重点污染源治理得到强化。文化、卫生、体育、新闻出版、广播电视、民族、宗教、外事、侨务、人防、气象、地震、民政、残联、老龄、档案、史志和妇女儿童等工作都取得新的成绩。

（七）精神文明和民主法制建设得到加强。五年来，我们认真学习邓小平理论和“三个代表”重要思想，积极开展向孔繁森同志学习活动和多种形式的精神文明创建活动，深入开展农村“三个代表”教育，人们的思想道德素质和文明程度不断提高。大力开展崇尚科学、反对迷信的教育，严厉打击了“法轮功”邪教组织。坚持依法治市、依法行政，普法教育扎实推进，村民自治制度全面推行。全民国防教育和双拥共建工作成效显著,被评为全省双拥模范城。加强廉政建设，深入开展反腐败斗争，查处了一批大案要案。加强社会治安综合治理，严厉打击各种犯罪活动，保持了政治安定和社会稳定。

（八）人民生活基本上达到小康水平。五年来，城乡居民收入有较大增长，物质生活不断改善。２００２年，全市城镇居民人均可支配收入达到6210元，农民人均纯收入达到2503元，分别比1997年增长55.8%和35.2%；城乡居民储蓄存款余额达到249.9亿元，比1997年增加107.8亿元，人均储蓄达到4451元。城镇居民和农民人均居住面积分别增加了2.92平方米和3.26平方米。汽车开始进入家庭，电话普及率达到每百人21.84部，人均预期寿命达到73岁。社会保障体系初步形成，“三条保障线”制度逐步完善，“两个确保”达到100%。认真做好就业和再就业工作，五年共安置12.2万名城镇劳动力就业，5.24万名下岗职工实现再就业，城镇登记失业率控制在４％以内，再就业率达到41%。大力实施劳务输出战略，全市在外务工人员达到５５万人。积极开展扶贫开发，贫困县乡实现了整体脱贫。

各位代表，回首五年的历程，我们可以自豪地说，过去的五年，是全市人民团结奋斗、高歌猛进的五年，是经济快速发展、综合竞争力迅速提高的五年，是发展环境明显改善、城乡面貌发生巨大变化的五年，是我们继续沿着中国特色社会主义道路胜利前进的五年！

五年来，聊城的发展变化是巨大的。取得上述令人鼓舞的成就，根本在于中央方针政策的正确指引，在于省委、省政府和市委的坚强领导，全市人民和社会各界都为聊城的发展付出了艰辛劳动，作出了不懈努力。在此，我代表市人民政府，向全市工人、农民、知识分子、各级干部和社会各阶层的建设者，向人民解放军、武警驻聊部队官兵和广大公安干警，向所有关心、支持聊城发展的国内外朋友们，表示衷心的感谢和崇高的敬意！

各位代表，回顾过去五年的工作，我们的主要体会是：第一，必须坚持解放思想、与时俱进，以思想的大解放促进经济的大发展。五年来，我们始终把解放思想、更新观念放在首位，持之以恒地开展了各种形式的解放思想教育活动，引导广大干部群众用市场经济的手段抓经济工作，用发展的办法解决前进中的问题，用创新的精神培植发展新优势，全市上下机遇意识、市场意识、开放意识、竞争意识明显增强，为加快发展提供了强大的思想动力和精神支持。第二，必须坚持从实际出发，确立正确的工作思路和发展战略。顺应形势发展的要求，立足聊城实际，围绕实现“加快发展跨入全省中等行列”的奋斗目标，确立了“突出五个重点，实现四个突破”的总体工作思路，全面实施了工业带动、外向带动、城市化、科教兴聊和可持续发展战略，并注重保持工作的连续性，在发展中不断探索创新，有效地推动了经济和各项事业的发展。第三，必须坚持突出重点、狠抓落实，集中力量突破影响全局的关键问题。把加大投入作为加快发展的根本措施，加大招商引资力度，加强银企联手，加快重点项目建设，有效地拉动了经济增长。把民营经济作为国民经济的重要组成部分，通过加大投入、培植骨干、建设园区、优化环境等有效措施，促进了民营经济的快速发展。坚持把中心城市作为区域经济发展的“龙头”，明确城市定位，加强规划建设管理，成功举办了首届“中国江北水城（聊城）文化旅游节”和“水城之秋观光周”活动，聊城的知名度和城市竞争力有了很大提高。紧紧抓住高新技术不放松，成功举办了“引进高校与科研院所科技成果洽谈会”和“科技成果洽谈月”活动，走出了一条符合聊城实际的高新技术产业发展路子。大力实施劳务输出战略，促进了农村剩余劳动力的有序流动和转移。第四，必须坚持大胆探索、勇于实践，全力推进改革开放。大力推进国有资产管理体制改革，积极开展全国农村税费改革试点，为全国全省提供了经验。面对入世的新形势，坚定不移地走出去，千方百计地引进来，大力开展招商引资和经贸洽谈活动，形成了全方位、多层次对外开放的新格局。第五，必须坚持两手抓、两手都要硬，正确处理改革发展稳定的关系。高度重视社会热点、难点问题，认真清理整顿企业高息集资和农村基金会，有效化解了可能出现的金融风险。积极做好就业和再就业工作，重视解决城乡群众的生活困难。切实加强信访工作，认真处理新形势下的人民内部矛盾。积极推行政务公开，推动政府工作“提速”，大力改善经济环境。按照“建设好班子，造就好队伍，树立好政风，争做好公仆”的要求，切实加强政府自身建设，公务员队伍素质不断提高。

各位代表，在肯定成绩的同时，我们也清醒地看到，发展中还存在不少困难和问题，工作中还存在许多缺点和不足。主要表现在：经济结构不够合理，整体素质不高，经济综合实力不强；农业基础仍然比较脆弱，产业化水平不高，农民收入增长比较缓慢；工业化水平比较低，技术创新能力不强，部分企业管理体制、运行机制还不能适应入世的要求；对外开放层次和水平较低，利用外资和外贸出口处于全省落后位次；科技对经济增长的贡献率较低，各类人才还十分匮乏；部分县市和乡镇财政困难，社会保障能力不强，就业和再就业压力较大，部分群众的生活比较困难；经济发展的软环境有待进一步改善，政府职能转变与市场经济发展还不相适应，一些行业不正之风和某些腐败现象仍不同程度地存在。认真克服和解决这些困难和问题，是全市各级政府的共同责任，是今后一个时期政府工作的重要任务，必须认真对待，切实抓好。

二、今后五年的目标任务

各位代表！党的十六大确立了本世纪头二十年全面建设小康社会的宏伟目标，向我们发出了实现中华民族伟大复兴的动员令。在新世纪、新阶段，聊城人民必须认清发展的趋势，跟上时代前进的步伐。上世纪后二十年，我们虽然取得了巨大成就，但与发达地区的差距仍然很大。本世纪头二十年，我们必须树雄心、立壮志，紧紧抓住这个大有作为的重要战略机遇期，加快推进现代化建设，努力实现跨越式发展，力争早日在山东西部崛起，向世人展示聊城卓越秀美、文明富裕的崭新形象，实现一代一代聊城人的不懈追求和美好夙愿。这是时代的要求、人民的意愿，是历史赋予我们的庄严使命。正是基于这一战略思考，市十次党代会提出了今后二十年的战略构想，即到2007年全市国内生产总值比2002年翻一番；2010年比2000年翻两番，与全省同步实现人均国内生产总值2万元的奋斗目标；再经过十年的努力，2020年比2010年再翻一番，人均国内生产总值达到5000美元左右，在全面建设小康社会的基础上，争取与全省同步提前基本实现现代化。并根据这一战略构想，提出了今后五年的总体目标：“跨越发展实现率先崛起，团结实干建设强市名城”。

各位代表！今后五年，是我市全面建设小康社会的重要阶段，是实现跨越发展、率先崛起的关键时期。政府工作总的要求是：坚持以邓小平理论和“三个代表”重要思想为指导，全面落实党的十六大、省八次党代会和市十次党代会精神，以跨越发展总揽全局，按照“三个坚持、三个关键、三个亮点、六个变化”的工作方针和“两个根本、三大优势、四大进程”的工作思路，解放思想，与时俱进，开拓创新，扎实苦干，全面完成“十五”计划和“十一五”计划前两年的任务，使全市经济发展跨上新台阶，改革开放实现新突破，人民生活质量提高到新水平，精神文明和民主法制建设取得新进展，各项工作开创新局面，加速聊城在山东西部率先崛起，为全面建设小康社会奠定坚实的基础。

今后五年的目标任务是：

－－综合经济实力显著增强。在优化结构和提高质量效益的基础上，国内生产总值年均增长１５％，达到750亿元，比2002年翻一番。努力保持经济与财政同步增长，力争地方财政收入突破４０亿元。

－－经济结构进一步优化。初步构筑起现代产业体系的基本框架，形成以加工制造业为主的十大百亿产业，以商贸旅游业为主的现代服务业，以优势农产品为主的现代农业，产业素质和市场竞争力明显提升，三次产业比例达到１５∶５２∶３３。

－－工业化、信息化、城市化水平明显提高。积极推进新型工业化，用信息化带动工业化，不断增强科技创新和可持续发展能力。大力推进城市化，加快建设江北水城，力争城市化水平达到40％左右。

－－社会主义市场经济体制进一步完善。市场取向的各项改革取得新突破，初步建立起符合社会主义市场经济体制要求的经济管理体制和运行机制。对外开放进一步扩大，经济国际化水平明显提高，力争实际利用外资突破3亿美元，出口创汇突破6亿美元。

－－全民的思想道德素质、科技文化素质和健康素质有较大提高。初步建成现代国民教育体系、科技和文化创新体系、全民健身和医疗卫生体系，创建学习型社会，大力发展文化事业和文化产业，促进人的全面发展和社会全面进步。

－－民主团结、生动活泼、安定和谐的政治局面进一步巩固和发展。加强社会主义民主建设，全面落实依法治市，人民的政治、经济、文化权益得到尊重和保障。社会秩序良好，形成团结、积极、向上的社会风尚。

－－人民的物质文化生活水平明显提高。努力增加城乡居民收入，不断提高物质生活质量。力争城镇居民人均可支配收入达到9000元以上，农民人均纯收入达到3500元以上。建立比较完善的社会保障体系，努力扩大劳动就业，认真解决弱势群体的基本生活保障，五年促进城镇就业18.5万人。

各位代表！全面完成今后五年的目标任务，我们必须按照“发展要有新思路，改革要有新突破，开放要有新局面，各项工作要有新举措”的要求，以解放思想为先导，把加快发展、结构调整、改革开放、保持稳定作为营造新优势、实现新突破的“重中之重”，切实把握好以下四点：第一，坚定不移地把加快发展作为第一要务。发展是硬道理。要实现率先崛起、建设强市名城的奋斗目标，就必须牢牢地把握发展这个主题，把全市上下的思想统一到跨越发展上来，切实增强机遇意识、忧患意识和竞争意识，自我加压谋跨越，千方百计求发展。要坚持用市场经济的手段抓经济发展，坚持用创新的精神培植发展新优势，只要效益好、结构新、可持续增长，能搞多快搞多快，不断提高发展水平，增强发展后劲。第二，坚定不移地把结构调整作为主线。面对日趋激烈的国内外市场竞争，要实现经济的跨越发展，必须站在参与国际分工与竞争的高度，立足当前，着眼长远，加快经济结构的战略性调整。要下大力调整产业结构，抓好优势农产品、百亿产业和现代服务业的发展，培育壮大优势突出、竞争力强的产业体系，努力增创竞争新优势。要继续调整所有制结构，毫无动摇地鼓励、支持和引导民营经济的发展。要统筹城乡发展，加快推进城市化，努力实现城乡经济的良性互动和协调发展。第三，坚定不移地推进改革开放。改革开放是调整经济结构、促进经济跨越发展的强大动力。要以创新的精神，不断改革与经济和社会发展不相适应的管理体制和运行机制，抓好国有资产管理体制改革和分配制度改革，健全现代市场体系和社会保障体系。坚持把招商引资作为经济发展的总抓手，全力实施“大开放、大招商，促进大发展”战略，在引进来的同时，坚定不移地走出去，大力发展对外贸易，积极参与国际竞争，加快经济国际化进程。第四，坚定不移地维护社会稳定大局。没有稳定的社会环境，改革和发展都无法顺利进行，已经取得的成果也可能丧失。要正确处理改革、发展、稳定的关系，正确处理新形势下的人民内部矛盾，切实解决好改革发展中出现的新情况、新问题，探索建立新形势下维护社会稳定的长效机制，确保在稳定的环境中推进改革发展，通过不断改革和发展保持社会稳定。

各位代表！今后五年的目标是宏伟的，任务是艰巨的，前景是美好的。我们坚信，有党的十六大精神的正确指引，有省委、省政府和市委的坚强领导，有历届政府奠定的良好工作基础，只要把全市人民的力量凝聚起来，把一切积极因素调动起来，聚精会神搞建设，一心一意谋发展，瞄准目标去赶超，就一定能够使聊城在山东西部率先崛起，就一定能够把聊城建设成为强市名城，让全市人民过上更加美好的生活！

三、 今后五年的工作重点

今后五年，改革开放和现代化建设的任务非常繁重。我们必须统筹安排，抓住重点，整体推进，着力抓好以下十个方面的工作。

(一)千方百计扩大投资，努力拉动经济快速增长。实现聊城的跨越发展，必须靠有效的、大规模的投入，必须把扩大投资作为长期的根本性措施，始终不渝地抓下去。今后五年，全市固定资产投资要保持较高增长，五年累计达到1200亿元以上。为此，要抓住一切机遇扩大投资，促进经济结构的调整和发展环境的优化，培植发展后劲。

全力抓好招商引资。把招商引资作为扩大投资规模、推动经济发展的总动力，进一步明确任务，落实责任，推动招商引资工作不断向纵深发展。要突出抓好大项目招商、园区招商和骨干企业产权招商，努力提高招商引资的层次和水平。继续把民营经济作为招商引资的重点，有针对性地到国内发达地区，特别是到民营经济发展快的地区招商引资，加强与他们的合作，承接他们的产业转移。

集中抓好重大项目建设。把工业经济、基础设施和城市建设作为投资的重点，全力抓好山东中华电力聊城发电厂二期２×６０万千瓦机组、聊城热电五期工程建设；抓好高唐时风集团重型汽车，信发希望、华信铝业公司、香港国资源投资公司电解铝，中通客车底盘，风祥集团１０万头奶牛、６０万亩牧草、６０万吨奶制品，聊城华润４０万纱锭，阳谷电缆集团光纤预制棒，冠洲集团２０万吨镀锌板带和２０万吨彩涂板，境内油气开发等重点工业项目建设；抓好南水北调工程聊城段、邯济铁路复线、黄河公路大桥、青银高速公路和德商高速公路聊城段以及公路干线网络等重大基础设施项目建设，确保按期竣工，尽早发挥效益。要认真研究国家的产业政策，积极做好新上项目的论证和报批，努力做到投产运营一批、开工建设一批、研发储备一批，保持投资的连续性，不断形成新的经济增长点。

完善扩大投资的内在机制。根据国家投融资体制改革的要求，充分发挥国有投资的引导作用，积极吸引和集中更多的社会资金投入经济建设。大力争取国债资金，保持国有投资快速增长。落实政策，放宽领域，加强服务，努力启动和扩大民间投资。充分发挥现有上市公司的作用，搞好配股和增发；加快培植上市资源，推进时风集团、泉林纸业、银河纸业、冠州集团、信发集团、凤祥集团等有条件的企业上市融资。要引导金融机构扩大信贷投放，全力支持地方经济建设。

（二）大力推进改革开放，进一步启动经济发展活力。面对经济全球化和入世的新挑战、新机遇，我们必须加快推进改革，提高市场化程度；全面扩大开放，提高经济的外向度，靠改革开放推动跨越发展。

深化市场取向的各项改革。一是深化国有资产管理体制改革。坚持管资产和管人、管事相结合，健全和完善三个层次的国有资产运营体系，积极探索有效的国有资产经营体制和方式，扩大国有资产运营体系的覆盖面，更好地实现政企分开，实现国有资产的保值增值。二是深化国有企业改革。以产权制度改革为突破口，采取国有股转让、合资合作、企业相互参股等多种形式，引导民间资本和外资参股控股国有企业，发展股份制和混合所有制经济，逐步实现投资主体多元化，加快建立现代企业制度步伐。积极推进国有大中型企业主辅分离、辅业改制，分流安置富余人员。进一步深化企业劳动、人事、分配制度改革，积极推行经营者年薪制、期权制、股权制。建立企业退出市场机制，加快劣势企业退出市场步伐。 三是深化农村改革。围绕减轻农民负担，增加农民收入，继续推进农村税费改革，完善配套政策，巩固扩大改革成果；按照“依法、自愿、有偿”的原则，建立土地流转制度，积极稳妥地推进土地规模经营；继续搞好林木、小型水利设施等农村产权制度改革；大力培植和发展农村各类合作经济组织，推进农业产业化经营。四是深化社会保障制度改革。全面落实“两个确保”和“一个低保”政策，扩大城镇养老、医疗和失业保险等社会保险覆盖面，加强社会保险费的征缴和支出管理。完善城镇居民最低生活保障制度，加快农村居民最低生活保障制度建设，切实保障低收入阶层和困难群体的生产生活。五是深化其它各项改革。坚持精减、效能、统一的原则，进一步推进机构改革，转变政府职能，集中解决行政管理体制存在的突出问题。加快土地管理和使用制度改革，政府高度垄断一级市场，积极培育和规范二级市场。

全面推进对外开放。坚持“引进来”和“走出去”相结合，以经济开发区和工业园区、重点骨干企业、民营企业、城市建设和服务业为重点，积极推进全社会对外开放。一是更大规模地利用外资。进一步拓宽利用外资领域，鼓励外商投资基础设施、现代农业、传统产业改造、高新技术和现代服务业。创新利用外资的方式，采取境外上市、企业并购、产权转让等形式，引导我市骨干企业与国际大公司、大财团进行产业对接，吸引跨国公司进行战略性投资合作，努力引进一批利用外资的大项目。继续实施走出去战略，坚持国外与国内相结合，大规模招商与小团组出击相结合，重点抓好对香港、台湾、韩国、日本以及国内外商投资密集区的招商引资。二是大力发展对外贸易。下大力调整出口产品结构，继续抓好肉食、蔬菜和纺织品出口，扩大电解铝、机电产品和纸制品出口，努力培植新的出口增长点。进一步加大扶持力度，抓好骨干企业的出口，培植一批出口大户。鼓励各类企业广泛开展与国外公司的合资合作，发展加工贸易，形成大进大出的新格局。加强企业间的联合与协作，利用优势企业聚合出口产品，带动相关企业产品的出口。实施市场多元化战略，巩固发展日韩和东南亚等传统市场，积极拓展俄罗斯、欧美、非洲、拉美、中东等新的市场。鼓励和支持有比较优势的企业对外投资，开展带料加工装配业务，带动商品出口和劳务输出。三是加快开发区和各类园区建设。加大对聊城经济开发区的扶持力度，加快基础设施和服务设施建设，深化体制改革和机制创新，努力营造良好的发展环境。聊城经济开发区要坚持以工业项目为主，以利用外资为主，以出口创汇为主，致力于发展高新技术产业，集中力量抓好招商引资特别是大项目的引进。力争用三年左右的时间，进入省级开发区中等行列；用五年左右的时间，达到一个中等县的经济规模，利用外资、出口创汇和高新技术产业的发展走在全市前列；用10年左右的时间，建成现代化的新城区。同时，抓好县（市、区）的各类园区建设。四是加强外经贸队伍建设。重点抓好政府外经贸干部队伍、企业外经贸人才队伍、开发区招商引资队伍、国内外招商代表队伍建设，为实现我市对外经贸工作的新突破提供强有力的人才支撑。

（三）坚持走新型工业化的路子，加快工业经济发展步伐。调整优化经济结构，全面建设小康社会，必须把发展工业摆在更加突出的位置，坚定不移地实施工业带动战略，着力推进工业结构的优化升级，确保工业经济更快更好的发展。

全力建设百亿产业，加快发展骨干企业。从今年开始到２０１０年，利用８年的时间，集中力量培植农用运输机械、汽车、电解铝及加工、造纸及纸加工、纺织及加工、医药、电力、油气化工、农副产品深加工和电子信息等１０个销售收入过百亿的大产业。每个产业培植2-3个核心企业，创造竞争优势，带动产业发展。要按照百亿产业发展规划，继续抓好“１０３０”工程，抓好重点骨干企业的发展，力争在五年内，培植起一批销售收入过３０亿、５０亿、核心竞争力强的大型企业集团。同时，抓好中小企业的发展，引导中小企业向配套型、加工型、服务型、科技型方向转变。

坚持以信息化带动工业化，推进工业结构优化升级。选择一批重点企业，建立信息技术应用示范工程和示范生产线，加快用信息技术武装工业。大力发展高新技术产业，集中力量扶持一批高新技术产业化项目，努力培植新的产业增长点。运用高新技术和先进适用技术，加快改造农用车、化肥、造纸、纺织、医药、机械配件等传统产业，进一步提高技术装备水平，培植一批名牌拳头产品。加快技术创新、管理创新和制度创新步伐，搞好企业研发中心建设，靠创新推动工业结构优化升级。

（四）大力推进农村产业结构调整，努力开辟农业发展和农民增收的新途径。全面建设小康社会，难点在农村，重点在农村，关键在农村。我们必须比任何时候更加关注农村、关心农民、支持农业。要以增加农民收入为中心，走调整结构、产业化经营、城乡统筹三位一体的发展路子。

进一步调整优化农业结构。今后五年，要继续压缩粮食面积，重点发展高效经济作物，粮经作物种植比例达到４∶６。大力培育优质小麦、蔬菜、畜禽、林果等优势农产品，推进区域化种植、规模化养殖、标准化生产，建成一批优质农产品生产基地，努力把优势农产品做大做强，提高我市农业的竞争力。以林纸结合项目和林果深加工项目为龙头，搞好绿色长廊、速生丰产林和经济林基地、城乡绿化和苗木基地建设，把林业这个产业做强做大。

加快推进农业产业化、标准化、国际化。采取对现有龙头企业进行扶持、与国内外有实力的大公司合资合作等方式，着力培植一批规模大、辐射带动能力强的农产品深加工龙头企业，努力提高农业产业化经营水平。大力发展农村中介服务组织和新型合作经济组织，提高农民的组织化程度。加快服务体系建设，建立和完善与国际接轨的农产品质量标准体系、检验监测体系、科技开发推广体系和卫生防疫体系，全面提高我市农产品的质量安全水平。

促进城乡经济协调发展。加快小城镇建设，引导二三产业向小城镇相对集中，促进农村劳动力合理有序流动。加强农业基础设施和生态环境建设，重点抓好位山灌区节水改造、金堤河二期治理、蓄水排涝工程和机井建设，大力推广节水技术，加快农村人畜吃水工程建设进度，提前一年完成农村饮水三年攻坚计划。加强农村公路建设，利用三年的时间实现所有行政村通油路。加大扶贫开发力度，加快脱贫步伐。切实搞好救灾解困工作。坚决制止“三乱”，减轻农民负担，保护农民利益。

（五）加快发展现代服务业，推动第三产业实现新跨越。今后五年，我们要把加快发展现代服务业作为推进经济结构战略性调整的重要内容，努力扩大总量，优化结构，提高在国民经济中的比重。一是大力发展现代物流业。要以现代信息技术为支撑，着眼于构筑现代化大流通的新格局，抓好香江光彩大市场等一批特色鲜明、辐射带动能力强的大型现代批发市场，努力增强综合服务功能，提高交易主体的组织化、交易商品的标准化和市场交易的现代化水平。要加快引进国内外知名的连锁经营企业，积极培育大型连锁企业集团，把新的物流业态引入聊城，形成新的经济增长点。要牢固树立大流通的观念，积极推进现代物流配送，加快建设现代化的物流服务平台。二是加快发展旅游业。今后五年，我们要以创建“中国优秀旅游城”为目标，进一步加大投入，加快旅游景点和配套设施建设，继续搞好“江北水城”的包装和促销，办好每年一度的“中国江北水城（聊城）文化旅游节”、“国际龙舟节”和“水城之秋观光周”活动，大力发展节庆假日经济，把聊城营造成国内旅游热点地区。三是抓好房地产、社区服务等行业的发展。注意把握房地产市场供求关系，搞好房地产发展的宏观调控，进一步规范和完善房地产市场，促进住宅业持续健康发展。大力发展卫生、家政、物业管理等社区服务业，使之成为新的经济增长点。四是积极发展教育培训、文化传媒、中介服务等需求潜力大的新兴产业，带动第三产业整体素质的提高。

（六）调整优化所有制结构，加快发展民营经济。毫不动摇地鼓励、支持和引导非公有制经济的发展，以“今后五年平均每年每个乡镇民营经济税收增加100万元、固定资产投资4000万元”为目标，进一步加大投入，激活民间投资，鼓励城乡居民采取多种形式创办民营企业。坚持扩大外延和提高内涵并重，鼓励民营企业引进先进技术和设备，发展高新技术产品，培植一批民营骨干企业、民营科技企业和民营出口企业。围绕农副产品加工、铝制品加工、轴承、农机配件等区域特色经济，大力发展民营企业群体，并把这些群体做大做强，形成地方支柱产业。把招商引资作为推动民营经济发展的主要措施，着力吸引一批大型民营企业来聊城投资创业。搞好民营经济园区建设，推动民营经济园区向特色园区、专业园区和科技型园区发展，增强园区的吸引力、带动力和保障力。进一步优化发展环境，少干预，多服务，少限制，多支持，放宽经营范围，降低创业门槛，为民营经济提供优质服务。

（七）加快城市化进程，促进县域经济实现新突破。把推进城市化作为重要战略，作为实现工业化的强大动力，形成城镇与农村、城市化与工业化相互促进、协调发展的格局。

全力抓好城市建设，提高城市综合竞争力。今后五年，要以建设卓越秀美的江北水城为总目标，优先发展聊城城区，加快发展临清市和６个县城，积极发展中心镇，把聊城创建成省级园林城、文明城、国家卫生城、中国优秀旅游城、国家环保模范城，努力实现“一年一变化，三年大变样”。按照高起点、高标准、出精品的要求，搞好城市规划，加快聊城中心区建设，着力打造江北水城。根据“一城五区”的总体布局，加快推进“东扩”、“南展”，拉大城市框架。突出抓好城市供热、供气、供水、排水、垃圾处理和道路等基础设施建设。加快城市绿化、水城开发和靓点工程建设，重点抓好古运河和徒骇河城区段开发、东昌湖开发配套、湖底隧道、古城开发等项目建设，完成江北水城主体框架的构筑。实施湖河沿岸建筑“平改坡”和“夜色景观”等美化工程，建设湖河沿岸风景线。抓好人民广场、文化广场、运河博物馆、凤凰剧场等城市标志性工程建设，提高聊城城市的层次和品位。围绕“湖、河、路、院”，建设组团式园林和绿化风景带，重点抓好城市出入口的绿化；继续搞好环城森林带和连接县市干线公路两侧各100米的绿色长廊建设。加快新时代广场、新东方广场、凤凰新城、柳泉花园、阳光花园等现代居住小区建设。坚持高效能管理城市，完善“两级政府、三级管理、四级网络”的城市管理体制，重点治理乱设摊点、乱扔垃圾、交通违章等现象，进一步规范城市秩序和城市居民行为，塑造良好的城市形象。加强社区建设，抓好村改居工作，进一步理顺城市社区管理体制。下大力抓好临清市、6个县城以及中心镇的建设，加快户籍管理制度改革，引导产业和人口向城镇集聚，提高城市化水平。积极推进城市经营，鼓励和吸引国内外投资者参与城市的建设和管理，逐步建立起多元化的投融资机制。全面推进国有土地储备制度建设，五年争取实现政府土地收益50亿元，不断增强城市自我发展能力。

坚持分类指导的原则，加快发展县域经济。把发展壮大县域经济摆在更加突出的位置，充分发挥县（市、区）的积极性和创造性，推动县域经济快速发展。支持基础好的县（市）加快膨胀经济规模，向全国、全省强县（市）迈进。高唐、临清、茌平要力争经过五年的努力，跨入全省乃至全国强县（市）行列。其它县区要瞄准先进，找准定位，制定赶超目标和措施，加快发展步伐，尽快缩小与强县（市）的差距。加大对困难县的扶持力度，高度重视困难乡、村的发展，帮助配好班子、选准路子，解决好制约发展的突出问题，使这些地方及早走上富裕之路。

（八）积极做好财税金融工作，千方百计扩大就业。大力开展增收节支，严格财政收支管理。认真落实鼓励产业和企业发展的有关政策，积极培植和壮大财源。拓宽聚财渠道，加强国有资产收益、土地收益、城市经营收益管理。依法加强税收征管，堵塞跑冒滴漏，做到应收尽收。按照建立公共财政的要求，规范财政支出范围，优先保证工资按时发放，重点安排社保资金和救灾解困资金，大力压缩一般性行政支出。进一步深化部门预算改革，完善政府采购制度，统筹安排预算内外资金，增强财政调控能力。牢固树立过紧日子的思想，勤俭办一切事业，严禁铺张浪费。积极落实信贷投放的各项政策，引导金融机构增加信贷投放，大力支持地方经济的发展。强化全社会的信用意识，建立健全符合市场经济要求的企业和个人信用体系，建设金融安全区。把扩大就业作为国民经济和社会发展的优先目标，贯彻市场就业的方针，千方百计扩大就业。市、县两级都要建立下岗失业人员贷款担保基金，积极开发公益性就业岗位，高度重视下岗失业人员中特困群体的再就业工作，深入开展以政策援助、岗位援助、就业服务援助、生活保障援助为主要内容的再就业援助行动。大力实施劳务输出战略，完善组织体系，树立聊城品牌，不断拓展劳务合作空间。引导全社会转变就业观念，鼓励自谋职业和自主创业。完善就业培训和服务体系，提高劳动者就业技能。

（九）大力实施科教兴聊战略，全面发展各项社会事业。发展科技教育事业，繁荣社会主义文化，提高人的素质，是经济发展和社会进步的基础和前提，必须把科技教育和社会主义文化建设放在更加突出的位置。

大力推进科技进步。深化科技体制改革，完善对科技人员的分配政策，充分调动科技人员的积极性；大力发展科技中介服务组织，搞活科技市场。以凤凰苑农业科技园为龙头，抓好农业科技园区建设，大力推广普及先进实用的农业新技术、新成果。加强企业技术中心建设，完善企业追求科技进步的动力机制，使企业真正成为技术创新的主体。以高新技术创业服务中心为依托，建立市级高新技术园区。继续加强与高等院校和科研院所的联合与合作，加快科技成果的引进、消化和吸收。充分发挥聊城大学和聊城职业技术学院在科技开发、成果转化、人才培养方面的辐射带动作用，积极推动聊城大学科技园的建设。加强信息基础设施建设，搞好信息资源的开发、整合和利用，加快电子政务建设，推进国民经济和社会信息化。

把教育事业摆在优先发展的战略地位。加大基础教育投入，巩固发展九年义务教育，加快发展高中阶段教育；加强教师队伍建设，全面推进素质教育，积极开展信息技术教育，不断提高教育质量。加强职业教育和培训，大力扶持高等职业教育，积极推进中等专业学校资源的优化配置。继续创造更加宽松的环境和条件，加快聊城大学和聊城职业技术学院的发展。鼓励和支持社会力量以多种形式办学。积极发展继续教育，构建终身教育体系。把人才建设作为重大的战略任务，改革用人制度，形成竞争上岗、优胜劣汰的用人机制，为各类人才脱颖而出创造条件；进一步完善政策，面向全国，面向世界，大力引进优秀的科技人才和经营管理人才来聊城创业。

全面落实三项基本国策。大力实施可持续发展战略，保持经济与人口、资源和环境的协调发展。继续把水污染和大气污染的防治作为重点，加强城市环境和区域性污染的综合整治，严格控制新污染源，坚持不懈地抓好企业污染治理工作。加强对资源的保护，提高资源的合理利用水平。积极发展节水农业、节水工业，建设节水型社会。进一步落实基本农田保护措施，合理调整城乡建设用地结构，节约利用土地。坚持和完善人口与计划生育目标管理责任制，认真贯彻落实《人口与计划生育法》和《山东省人口与计划生育条例》，建立和完善“依法管理、村（居）民自治、优质服务、政策推动、综合治理”的新机制，稳定低生育水平，提高人口素质。

加快发展文化、卫生、体育等其它社会事业。深入挖掘聊城的历史文化内涵，大力吸收当代的先进文化，努力培育“自强不息、务实创新、大气开放、诚实守信”的聊城人文精神。进一步整顿和规范文化市场，繁荣文艺创作，搞好文物保护。加强博物馆、图书馆、文化馆、艺术馆等文化基础设施建设，发展各类群众文化。坚持正确的舆论导向，加强新闻出版、广播电视、网络等信息传媒的建设和管理。积极推进城镇卫生改革，强化社区卫生服务，全面加强农村卫生工作，建立以大病统筹为主的新型合作医疗制度和医疗救助制度，进一步提高人民群众的健康水平。加强体育场、体育馆等体育设施建设，重视体育产业的发展，抓好竞技体育，深入开展全民健身运动，提高全民健康水平。进一步做好档案、史志、对台、民族、宗教、外事、侨务、气象、防震、救灾、老龄以及残疾人等项工作。

（十）加强政治文明和精神文明建设，努力保持社会稳定。扩大社会主义民主，健全社会主义法制，建设社会主义精神文明，是推动经济建设和各项社会事业发展的基本要求。必须在搞好经济建设的同时，加强政治文明和精神文明建设。

加强社会主义政治文明建设。坚持人民代表大会制度，依法向人大及其常委会报告工作，认真执行和落实有关决议、决定，自觉接受监督。加强与政协的联系与沟通，积极支持政协履行政治协商、民主监督、参政议政的职能。高度重视人大代表建议和政协委员提案办理工作，主动听取民主党派、工商联、无党派民主人士和各人民团体的意见和建议。扩大基层民主，健全村民自治、城市居民自治制度，完善职工代表大会和其它形式的企事业民主管理制度。坚持依法治市，深入开展“四五”普法教育，加强法律援助工作，提高全民法律意识和全社会的法制管理水平。

加强社会主义精神文明建设。深入开展党的十六大精神的宣传教育，用邓小平理论和“三个代表”重要思想武装广大干部群众，不断提高贯彻执行党的基本理论、基本路线、基本纲领的自觉性和坚定性。全面贯彻《公民道德建设实施纲要》，弘扬爱国主义精神，以为人民服务为核心，以集体主义为原则，以诚实守信为重点，加强社会公德、职业道德、家庭美德教育。继续开展群众性精神文明创建活动，提高文明城市、文明村镇、文明行业的创建工作水平。普及科学知识，倡导科学精神，反对封建迷信，提高全民科学素养。加强国防后备力量建设和民兵预备役工作，搞好全民国防教育和人民防空工作，做好军转干部、城镇退伍士兵安置和拥军优属工作，增进军政军民团结，争创全国双拥模范城。

全力维护社会稳定。认真落实《聊城市安全生产管理办法》，强化安全生产责任制，加大专项整治力度，坚决杜绝各类重大恶性事故的发生。继续下大力整顿和规范市场经济秩序，创造各类市场主体平等竞争的市场环境，依法严厉打击制售假冒伪劣商品和偷税漏税、逃税骗汇、走私贩私等不法行为。继续开展严打整治斗争，依法严厉打击各种犯罪活动，防范和惩治邪教组织的犯罪活动，坚决扫除社会丑恶现象。坚持打防结合、预防为主，落实社会治安综合治理的各项措施，健全治安防范工作机制，保持良好的社会秩序。认真做好信访工作，进一步健全和完善人民调解制度，保持安定团结的局面。

四、努力做好２００３年的工作

各位代表！全面贯彻党的十六大精神，圆满完成今后五年的任务，至关重要的是开好头、起好步，努力做好今年的工作。建议今年经济社会发展的预期目标为：国内生产总值增长１５％，力争增长１６％；地方财政收入增长１８％，力争增长２０％，总量突破２０亿元；全社会固定资产投资增长３０％以上，力争总量突破２００亿元；外贸出口增长５０％以上，力争总量达到２.５亿美元；实际利用外资增长５０％以上，力争总量突破１亿美元；社会消费品零售额增长１５％；农民人均纯收入增长６％；城镇登记失业率控制在４％以内；人口自然增长率控制在6.5‰以内。实现这些任务目标，我们必须把握好以下几个方面。

（一）要抓住两个关键。一是千方百计扩大投资。全年重点抓好茌平电解铝及配套热电机组、山东中华电力聊城发电厂一期2号机组、聊城热电四期工程、聊城华润10万纱绽等14个项目建设，确保按期竣工，尽早发挥效益。切实抓好招商引资任务的落实，着力突破一批利用内外资的大项目。二是大力推进结构调整。进一步调整优化农村产业结构，确保粮经作物种植比例达到5∶5，林牧业占农业总产值的比重有明显提高；坚定不移地实施工业带动战略，突出抓好高新技术和先进适用技术的推广应用，促进工业结构优化升级；大力发展以商贸、旅游为重点的现代服务业；继续实施城市化战略和劳务输出战略，引导更多的农村劳动力向城镇和市外转移。

（二）要营造三个亮点。一是切实抓好外经外贸。坚持“引进来”和“走出去”相结合，进一步拓宽利用外资领域，力争在引进利用外资的大项目上实现突破，在现代服务业、高新技术、基础设施利用外资上实现突破；调整出口产品结构，培植出口大户，开拓国际市场，努力扩大对外贸易。二是切实抓好高新技术产业。要大力扶持高新技术产业，认真组织好“科技合作回访周”活动，争取引进更多的科技成果，提高高新技术产业的比重。三是切实抓好民营经济。要加大投入，培植骨干，发展特色产业，加快园区建设，促进民营经济快速健康发展。

（三）要突出四个重点。一是组织实施好百亿产业工程。搞好总体规划和专项规划，抓好“1030”工程，做大做强工业经济，进一步提高工业比重，提高工业经济的效益和质量。二是大力发展服务业。抓好香江光彩大市场等商贸设施建设，推动商贸流通上规模、上水平；精心组织好江北水城文化旅游节等节庆活动，拉动旅游业快速发展。三是加快推进城市化。继续构筑江北水城的主体框架，抓好城市标志性工程建设，加强城市管理，改善城市环境，提升城市的品位和形象。精心搞好城市资产的经营，盘活存量，增加政府收益，增强自我发展能力。四是深化国有企业改革。积极稳妥地推进国有、国有控股企业面向国内外出让产权股权，实现投资主体多元化；推行企业经营者年薪制、期权制、股权制，充分调动经营者的积极性。

（四）要解决好五个问题。一是切实做好财税金融工作。大力开展增收节支，确保财政收支平衡。严格按照保工资、保重点、保法定支出的顺序安排支出，严禁铺张浪费。加快金融安全区建设，优化金融运行环境。二是切实做好社会保障工作。进一步完善社会保障体系，全面落实“两个确保”和“一个低保”政策，做到应保尽保。三是切实做好就业和再就业工作。加快市县两级下岗失业人员贷款担保基金建设，积极开发公益性岗位，最大限度地安置下岗失业人员。高度重视下岗失业人员中特困群体的再就业工作。四是切实抓好安全生产。搞好安全生产的宣传教育，进一步强化基层和基础工作，开展安全生产专项整治，确保我市安全生产形势的稳定。五是进一步优化经济发展环境。深入开展“优化环境年”活动，认真解决环境方面存在的突出问题，全面建设“信用聊城”，全力营造“引商、亲商、尊商、留商”的社会环境，努力创造环境新优势。

五、 进一步加强和改进政府工作

新世纪、新阶段、新目标对政府工作提出了更新更高的要求。各级政府必须解放思想，与时俱进，按照“建设好班子，造就好队伍，树立好政风，争做好公仆”的要求，以“从严治政，狠抓落实”为主线，加强学习，转变职能，改进作风，把各级政府建成廉洁、高效、求实、创新的政府，建成干事创业、为民谋利的政府。

（一）加快建设学习型政府。党的十六大提出了建设学习型社会的目标，各级政府要走在全社会的前列，树立终身学习的观念，努力建设学习型政府。要在全市政府机关开展“创建学习型机关，建设学习型队伍”活动，提高政府工作人员的素质。要认真学习邓小平理论和“三个代表”重要思想，保持政治上的坚定性。要适应新形势、新任务的要求，不断学习新知识，增长新本领。要坚持理论联系实际的学风，大力加强调查研究，深入基层了解实际情况，及时研究解决改革和建设中的新情况、新问题，不断提高驾驭市场经济的能力和应对复杂局面的能力。

（二）全面提高工作效率。按照社会主义市场经济的要求，积极推进政企分开、政事分开，把政府职能真正转变到宏观调控、市场监督、社会管理和公共服务上来。按照政府“提速”的要求，继续深化行政审批制度改革，加快推进电子政务建设，办好市政府行政审批服务大厅，进一步完善工作机制，简化办事程序，提高办事效率。全面推行政务公开，自觉接受人民群众的监督。大力推进依法行政，加强行政执法监督，建立健全行政执法责任制、评议考核制和错案责任追究制，积极稳妥地在城市开展相对集中行政处罚权工作，进一步规范行政执法行为。

（三）切实改进工作作风。按照“八个坚持，八个反对”的要求，牢记党的宗旨，树立群众观念，把群众利益作为工作的根本出发点和落脚点。要大力精简文件，压缩会议，减少评比达标活动，力戒形式主义、官僚主义。要深入基层，深入群众，关心群众疾苦，认真解决人民群众特别是困难群众的生产生活问题。要埋头苦干，狠抓落实，努力做到不空谈、重实干，不松懈、抓紧干，不敷衍、用心干，不掣肘、同心干。干就要干好，做就要做成。凡是定了的事情、看准了的事情，都要坚决地抓紧、抓实、抓好，切实抓出成效。要建立健全行政效能监察监督制度，对政府机关履行职责的情况定期进行评议，评议结果作为衡量部门政绩的主要依据。

（四）努力保持清正廉洁。要从严治政，加强对政府工作人员的教育和管理，筑牢拒腐防变的思想道德防线。要深化改革，创新体制，进一步加大从源头上预防和治理腐败的力度。要深入开展反腐败斗争，严肃查处违纪违法案件，维护党纪国法的严肃性。全面落实廉政责任制，对违反责任规定的领导干部，必须进行责任追究。严格执行中央、省和市委关于廉政建设的各项规定，做到廉洁自律、率先垂范，树立良好的政府形象。

各位代表！我们已经踏上全面建设小康社会的新征程。聊城的前程充满希望，我们的事业任重道远。我们一定加倍努力地工作，以高昂的斗志锐意改革，以饱满的热情致力创新，以求实的精神促进发展，用优异的成绩向全市人民交上一份合格的答卷。让我们高举邓小平理论伟大旗帜，全面贯彻“三个代表”重要思想，更加紧密地团结在以胡锦涛同志为总书记的党中央周围，在省委、省政府和市委的正确领导下，解放思想，与时俱进，团结一心，开拓前进，为实现率先崛起、建设强市名城，为全面建设小康社会、创造更加美好的未来而努力奋斗！
